# Spin transport in polarization induced two-dimensional electron gas channel in *c*-GaN nano-wedges

**DOI:** 10.1038/s41598-021-84451-y

**Published:** 2021-03-05

**Authors:** Swarup Deb, Subhabrata Dhar

**Affiliations:** Department of Physics, Indian Institute of Technology Bombay, Powai, Mumbai, 400076 India

**Keywords:** Two-dimensional materials, Spintronics

## Abstract

A two-dimensional electron gas (2DEG), which has recently been shown to develop in the central vertical plane of a wedge-shaped *c*-oriented GaN nanowall due to spontaneous polarization effect, offers a unique scenario, where the symmetry between the conduction and valence band is preserved over the entire confining potential. This results in the suppression of Rashba coupling even when the shape of the wedge is not symmetric. Here, for such a 2DEG channel, relaxation time for different spin projections is calculated as a function of donor concentration and gate bias. Our study reveals a strong dependence of the relaxation rate on the spin-orientation and density of carriers in the channel. Most interestingly, relaxation of spin oriented along the direction of confinement has been found to be completely switched off. Upon applying a suitable bias at the gate, the process can be switched on again. Exploiting this fascinating effect, an electrically driven spin-transistor has been proposed.

## Introduction

There are different proposals for electrical control of spin current in semiconductor structures^1–4^. Narrow bandgap semiconductors with large coupling (SOC) can serve as the medium for spin manipulation. However, strong SOC in such systems enhances spin–flip rate by mixing the spin states of the conduction band, which makes the propagation of spin over a sufficiently long distance a challenge^5^. Dissipationless flow of spin over long distance is an important requirement for the development of spin-based logic circuits^6,7^. The key approach is to use narrow bandgap semiconductors for spin control and low SOC semiconductors as link^5,8,9^. GaN, a wide bandgap semiconductor with weak coupling, has received overwhelming attention in recent times. Spin relaxation mechanism has been studied extensively in GaN bulk^10,11^, quantum wells^12,13^ and nanowires^14–17^. Notably, GaN nanorod based spin-lasers with polarization of about $28\%$ has been demonstrated at room temperature^18^. In fact, single-crystal GaN nanostructures are found to show much longer spin diffusion length as compared to bulk. Spin-valve effect with a spin relaxation length of $\sim 260$ nm has been demonstrated in a single GaN nanowire at room temperature^16^. Interestingly, an even longer spin diffusion length of $\sim 1\,\upmu {{\text {m}}}$ has been observed at room temperature in GaN nanowires with triangular cross-section^15^.

Recently, we have shown the formation of 2DEG in the central vertical plane of a wedge-shaped *c*-oriented GaN nanostructure. In wurtzite GaN, the asymmetric placement of positively charged ${{\text {Ga}}}^{3+}$ planes between negatively charged ${{\text {N}}}^{3-}$ planes (equivalently vice-versa) gives rise to a net dipole moment in a unit cell, leading to a spontaneous charge polarization along *c*-direction in the crystal^19,20^. Spontaneous charge polarization (henceforth referred as spontaneous polarization) along *c*-direction in Ga-polar GaN leads to the build-up of negative polarization charges at both the inclined facades. Electrons in the conduction band (for an n-type material) thus experience Coulomb repulsions from both the negatively charged facades, which has been shown to result in 2D confinement of the carriers in the central plane of the wedge^21,22^. The study further predicts remarkably high electron mobility in this channel, which arises due to the natural separation of the electrons (in the middle) from the ionized donors (at the boundaries)^21^. These predictions are also supported by the experimental findings of high conductivity^23–26^ and long phase coherence length^24,27,28^ for electrons in networks of *c*-oriented GaN nano-wedges. Since the 2D confinement takes place deep inside the structure, the symmetry between the conduction and valence band is intact over the entire potential profile. This contrasts with the case of 2DEG formed in heterojunctions, where the symmetry is broken due to band offset at the interface. This broken symmetry introduces an additional term in the Hamiltonian due to the Rashba effect. The 2DEG, in the present case, is thus unique in the sense that it is naturally protected from any geometry driven Rashba field. This has motivated us to understand spin transport in this system.

Here, we have theoretically studied electron spin relaxation via D’yakonov-Perel’ (DP) mechanism in the 2DEG channel formed in wedge-shaped *c*-oriented GaN nanowalls. Since electron mobility in this system is expected to be significantly high and the wurtzite lattice is non-centrosymmetric, DP is likely to dominate the relaxation process^29^. The kinetic equation for spin density of the conduction band electrons is numerically solved to obtain the relaxation times associated with spin projections along different crystalline directions as a function of donor concentration and gate bias. Relaxation rate is shown to be a strong function of not only the spin-orientation but also the density of the carriers in the channel. Most notably, the relaxation of spin oriented along the confinement direction is found to be completely shutdown. Interestingly, the phenomenon is unaffected by any deviation from the symmetrical shape of the structure. Spin relaxation can again be switched-on by increasing electron concentration in the channel through gate bias. These findings lead us to propose a spin-transistor device.

## Theory

**Figure 1:**
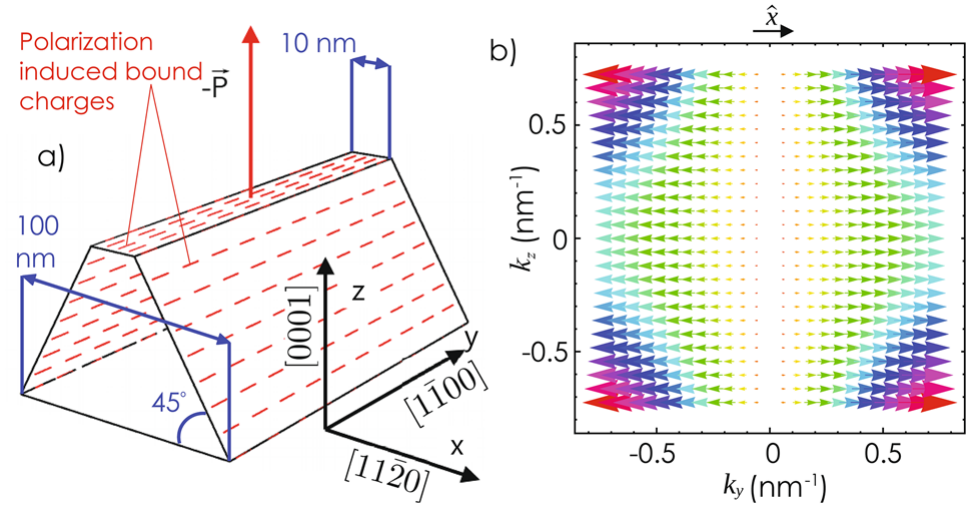
(**a**) Schematic representation of the nanowedge structure. Growth [0001] and confinement [$11{\bar{2}}0$] (*a*-axis) directions are regarded as *z* and *x*-axes, respectively. (**b**) Arrows represent the direction and magnitude of the effective field ${\mathbf {\Omega }}({\mathbf {k}})$ seen by the conduction band electrons with wave-vector ${\mathbf {k}}$ in the 2DEG channel.

Spontaneous polarization, ${\mathbf {P}}$ along $-{\hat{z}}$ induces a net negative polarization charge (in case of Ga-polar GaN) of density $\rho _s = {\mathbf {P}}\cdot {\hat{n}}$, where ${\hat{n}}$ is the unit vector normal to the surface, on the inclined facades of a wedge-shaped wall structure, as shown schematically in Fig. 1a. In case of n-type GaN nanowalls, polarization charges on the side facades can create a repulsive force to the conduction band electrons resulting in a confinement in the central vertical ($11{\bar{2}}0$) plane of the nanowall^21^. Polarization charges at the bottom surface are often compensated/suppressed by the charges of opposite polarity resulting from the substrate polarization.

Conduction band minimum for wurtzite (WZ) GaN remains spin degenerate even after considering the effects of crystal field and SOC^30^. Lack of inversion symmetry in the WZ lattice results in a ${\mathbf {k}}$ dependent term in the Hamiltonian, which can be expressed as^5,9–12,14,31,32^:

1 $$\begin{aligned} H_{SO}({\mathbf {k}})=\{\alpha _R+ \beta _D (b_D k_z^2-k_x^2-k_y^2)\}(k_y \sigma _x -k_x \sigma _y ) \end{aligned}$$

where $\alpha _R$ determines the strength of the *k*-linear Rashba like contribution. This term arises in bulk (even in the absence of structural inversion asymmetry) as a result of the built-in electric field due to spontaneous polarization^12^. $\beta _D$ and $b_D$ are the Dresselhaus parameters associated with the $k^3$-terms. In case of a 2DEG confined along [11${\bar{2}}$0] direction (*x*-axis), one can get an expression for $H_{SO}$ for the conduction band electrons by replacing $k_x$, $k_x^2$ terms in Eq.( 1) by their expectation values^32–35^. Note that $\langle k_x \rangle =0$ for bound eigenstates. $H_{SO}$ can thus be expressed as:

2 $$\begin{aligned} H_{SO}({\mathbf {k}})=\{\alpha _R+ \beta _D (b_D k_z^2-\langle k_x^2 \rangle -k_y^2)\}k_y \sigma _x \end{aligned}$$

$H_{SO}$ can also be expressed as $H_{SO}({\mathbf {k}})=\frac{\hbar }{2}{\mathbf {\Omega }} ({\mathbf {k}})\cdot \mathbf {\sigma }$, where ${\mathbf {\Omega }}({\mathbf {k}})$ represents an effective magnetic field and $\mathbf {\sigma }$ is the electron-spin. In bulk WZ-GaN, ${\mathbf {\Omega }}$ always lies in *xy* plane (Eq. 1) and its orientation is decided by the magnitude of $k_x$ and $k_y$. Interestingly, when 2DEG is formed in ($11{\bar{2}}0$)-plane, the effective magnetic field is always along ${\hat{x}}$ (+ or −) direction irrespective of the magnitude and orientation of the in-plane wave-vector ${{\mathbf k}_{\mathbf {\parallel}}}$, as shown in Fig. 1b. However, the magnitude of ${\mathbf {\Omega }}$ depends upon the *y*- and *z*-components of ${{\mathbf k}_{\mathbf {\parallel}}}$. Below we will see that it has a remarkable consequence on the DP spin relaxation properties of the 2DEG in this case.

**Figure 2:**
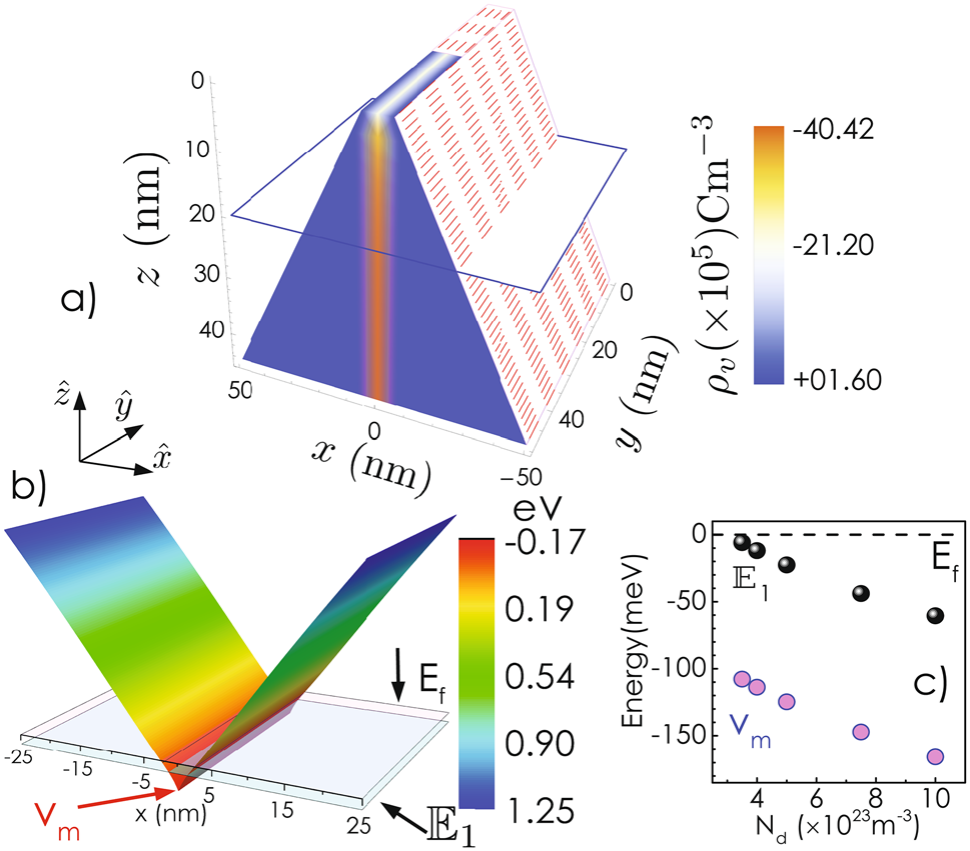
(**a**) 3D color plot for the charge density $\rho _v(x, y, z)$ inside the wedge-shaped *c*-oriented WZ-GaN wall. Tip of the wall has intentionally kept half-uncovered to show the extent of charge distribution along the *y*-axis. (**b**) A 3D-plot for $E_c$ in *yz*-plane obtained by solving 2D-Poisson’s equation. (**c**) Variation of the ground state energy eigenvalue and the depth of the potential well with the donor concentration, $N_d$.

The D’yakonov-Perel’ spin relaxation equation for the density, $S_i(t)$ of the spin projected along ${\hat{i}}$ (where, $\,i=x,y,z$) can be written as^34,36–38^ ${\dot{S}}_i(t)=\sum _{-\infty }^{\infty }\frac{\int _{0}^{\infty }d{\mathcal {E}}({\mathbf {k}}_{\parallel })\delta f\tau _nTr([H_{-n},[H_n,\sigma _j]]\sigma _i)}{-2\hbar ^2\int _{0}^{\infty }d{\mathcal {E}}({\mathbf {k}}_{\parallel })\delta f}S_j(t)$ where $\delta f$ $=$ $(f_+-f_-)$, $f_{\pm }$ are the Fermi distribution functions for electrons with spin $\pm 1/2$. $\tau _n^{-1}(k_{\parallel })=\frac{{\mathcal {A}}}{4\pi ^2}\int _{0}^{2\pi }{\mathcal {S}}({\mathbf {k}}_{\parallel },{\mathbf {k}}^{\prime }_{\parallel })[1-cos(n\theta )]d\theta$, where ${\mathcal {S}}({\mathbf {k}}_{\parallel },{\mathbf {k}}^{\prime }_{\parallel })$ represents spin independent momentum scattering rate between ${\mathbf {k}}_{\parallel }$ and ${\mathbf {k}}^{\prime }_{\parallel }$ , $\theta$ is the angle between the initial and final wave-vectors, ${\mathcal {A}}$ is the box normalization factor for the free part of the wavefunction of the confined electrons and $H_n=\int _{0}^{2\pi }\frac{d\phi }{2\pi }H_{SO}e^{-{\mathtt {i}} n\phi }$.

It can be shown that ${\dot{S}}_x(t)=0$ [see “Supplementary”], which implies that the DP mechanism does not alter the spin projection along ${\hat{x}}$ i.e., the relaxation time for *x* component of spin $\tau ^s_x$ is infinite. This can also be understood from the following perspective. Since $H_{SO}$ always commutes with $\sigma _x$, $S_x$ remains a good quantum number irrespective of the direction and magnitude of ${\mathbf {k}}_{\parallel }$. Note that the statement is valid when all other effects which can cause a spin mixing are neglected. Our calculations further show that the relaxation times for *y* and *z* spin components, which follow $1/\tau ^s_i=-{\dot{S}_i(t)}/{S_i(t)}$ [$i=y,z$], are the same and can be expressed as (see “Supplementary” for detailed derivation): $\frac{1}{\tau ^s_{y,z}}=\frac{8}{2\hbar ^2}\left[ \sum _{-1,1} (C_1 k_{\parallel }+C_2 k_{\parallel }^3)^2\tau _n+\sum _{-3,3} C_3^2 k_{\parallel }^6\tau _n\right]$ where $C_1=(\alpha _R-\beta _D\langle k_x^2 \rangle )/2$, $C_2 = \beta _D (b_D-3)/8$, and $C_3$=$-\beta _D (b_D+1)/8$ are material dependent constants. This is consistent with the fact that under the DP mechanism $\tau _z^{-1} = \tau _x^{-1} + \tau _y^{-1}$. Therefore, $\tau _z = \tau _y$ is expected with $1/\tau _x = 0$. Note that, the phenomena of vanishing spin relaxation has also been reported earlier in various contexts^38–40^, however, their origin is entirely different than what we have discussed here. It should be noted that the inclusion of higher order terms [$\sim {{\text {O}}}(k^4$) and higher] in the expression of the Hamiltonian (Eq. 1) may in principle contribute to the spin relaxation. However, their contribution is expected to be negligible as the Fermi wave-vector ($k_f$) even for the 2D electron concentration of $10^{17}\;{{\text {m}}}^{-2}$ in the well is sufficiently close to the conduction band minimum at the $\Gamma$-point^40^.

## Summary of main results and discussions

As a test case, we have considered a wedge-shaped *c*-oriented WZ-GaN nanowall with a background donor concentration ($N_d$) of $1\times 10^{24}\,{\text {m}}^{-3}$ and dimensions as shown in Fig. 1a. The volumetric charge density $\rho _v(x,y,z)$ and the conduction band minimum, $E_c(x,y,z)$ have been obtained by solving two dimensional (2D)-Poisson’s equation with appropriate boundary as well as charge neutrality conditions as described in Ref.^21^. Note that symmetry of the problem ensures that $\rho _v(x, y, z)$ and $E_c(x,y,z)$ are invariant along *y*-axis. $\rho _v(x,y,z)$ is shown in Fig. 2a. Formation of 2DEG is evident from the figure. In order to find the bound energy eigenstates, one needs to solve the 2D-Schrödinger equation on *xz* plane. However, due to the weak dependence of $E_c(x,z)$ on *z*, the 2D-Schrödinger equation can be approximated as a set of one dimensional (*x*-dependent) Schrödinger equations each of which is associated with a specific *z* position^41^. These calculations are carried out at $T=10$ K. Figure 2b shows the conduction band profile [$E_c(x,y)$] at a depth of 20 nm from the tip. Evidently, the central part of the $E_c(x)$ profile goes below the Fermi surface ($E_f$), forming a trench that extends along the *y*-direction. ${\mathbb {E}}_1$ denotes the first energy eigenstate of the quantum well at that depth. We have extended the calculation for several other $N_d$ values. In Fig. 2c, ${\mathbb {E}}_1$ at $z=20$ nm and the depth of the well (${\text {v}}_{\text {m}}$) with respect to the Fermi energy are plotted as a function of $N_d$ . Evidently, the separation between ${\mathbb {E}}_1$ and $E_f$ (also ${\text {v}}_{\text {m}}$ and $E_f$) increases monotonically with increasing $N_d$, which can be directly attributed to the increasing free carrier density in the 2D channel with increasing donor concentration. It should be mentioned that the range of the donor concentration is chosen in a way that only one eigenstate exists around the Fermi level, at that depth from the wall apex. Henceforth, we have shown the calculations only for the electrons lying at a depth of 20 nm from the tip of the wall.

**Figure 3:**
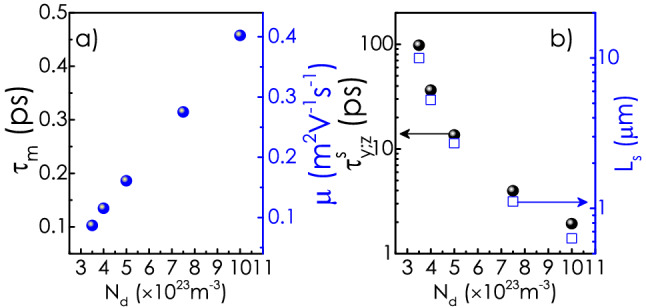
(**a**) Variation of momentum relaxation time ($\tau _m$), mobility ($\mu$), (**b**) relaxation time for *y* and *z* component of spin($\tau ^s_{y,z}$) and corresponding spin coherence length ($L_s$) as a function of $N_d$. All the calculations are carried out at ${\text {T}}=10$ K.

Next, we calculate momentum relaxation time, $\tau _m$ (the details of these numerical calculations can be found in Ref.^21^) of the quantum confined electrons limited by the neutral donor scattering, which plays the most significant role in deciding the electron mobility at low temperatures in this system^21^. It should be noted that we have considered a spherically symmetric hard wall potential profile for the neutral impurities to calculate the scattering cross-section and $\tau _m$, subsequently. Variation of $\tau _m$ and mobility ($\mu$) (in right *y*-ordinate) with $N_d$ is plotted in Fig. 3a, which clearly shows an increase of $\tau _m$ with the donor concentration. The effect can be attributed to the increasing separation between ${\mathbb {E}}_1$ and $E_f$ [i.e., the increasing electron density] with $N_d$. An increase of the separation leads to the enhancement of the electron’s kinetic energy, which results in a lower scattering cross-section. Relaxation time for *y* and *z* components of spin $\tau ^s_{y,z}$ as a function of $N_d$ is shown in Fig. 3b. As expected, the spin relaxation time decreases as $\tau _m$ increases. It should be noted that $\tau ^s_{y,z}$ comes out to be $\sim 100$ ps for the nanowall with $N_d=0.35\times 10^{18}\,{\text {cm}}^{-3}$. Interestingly, a few factor change in donor density alters the spin relaxation time by about two orders of magnitude. The spin coherence length, $L_s$ = $\tau ^s v_f$, where $v_f$ is the Fermi velocity, is also plotted as a function of $N_d$ in the same panel. Note that $L_s$ for the lowest donor concentration comes out to be as high as 10 $\upmu$m.

**Figure 4:**
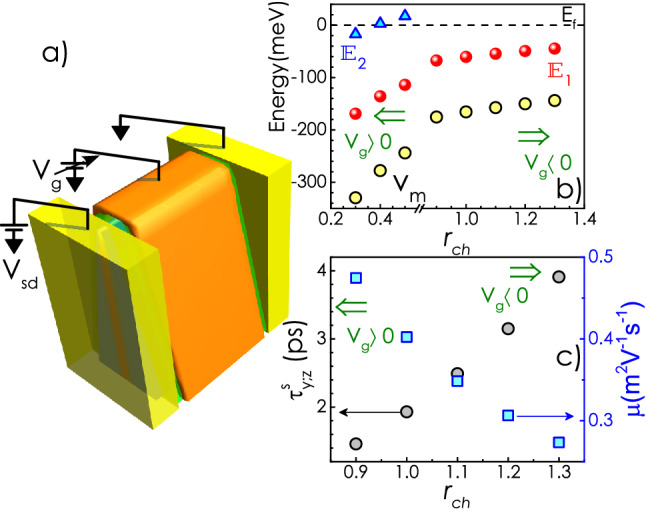
(**a**) Schematic picture of the gated device. (**b**) Variation of the energy eigenvalues and the depth of the potential well with $r_{ch}$. (**c**) Electron mobility ($\mu$) and $\tau ^s_{y,z}$ as a function of $r_{ch}$. All the calculations are carried out at ${\text {T}}=10$ K.

One way to manipulate spin transport in this system is to control the carrier concentration in the channel through gate bias. The idea is that with increasing carrier concentration, the kinetic energy of the electrons around the Fermi level increases. This, in turn, can change both $\mu$ the electron mobility and $\tau ^s_i$ the spin relaxation time. To calculate these changes, one needs to incorporate the effect of gate voltage in the Poisson equation solution. Gate contact and the semiconducting channel together form a capacitor [see Fig. 4a]. When the source and drain electrodes are grounded, and a positive(negative) gate voltage is applied, some amount of electrons are pumped(removed) into(from) the channel by the power supply. Since the semiconductor is no longer charge neutral, the Poisson’s equation has to be solved by satisfying appropriate positive to negative charge ratio condition (instead of satisfying charge neutrality) to obtain the $E_c$ profiles. The total positive to negative charge ratio ($r_{ch}$) should be less(greater) than 1 when sufficiently positive(negative) gate voltages are applied. The following calculations are done with $N_d= 1\times 10^{24}\,{\text {m}}^{-3}$. As shown in Fig. 4b, the gap between ${\mathbb {E}}_1$ and $E_f$ decreases as $r_{ch}$ increases. Note that when $r_{ch}$ is sufficiently less than unity [high $(+)ve$ gate voltages], more than one bound states are formed below the Fermi level.

**Figure 5:**
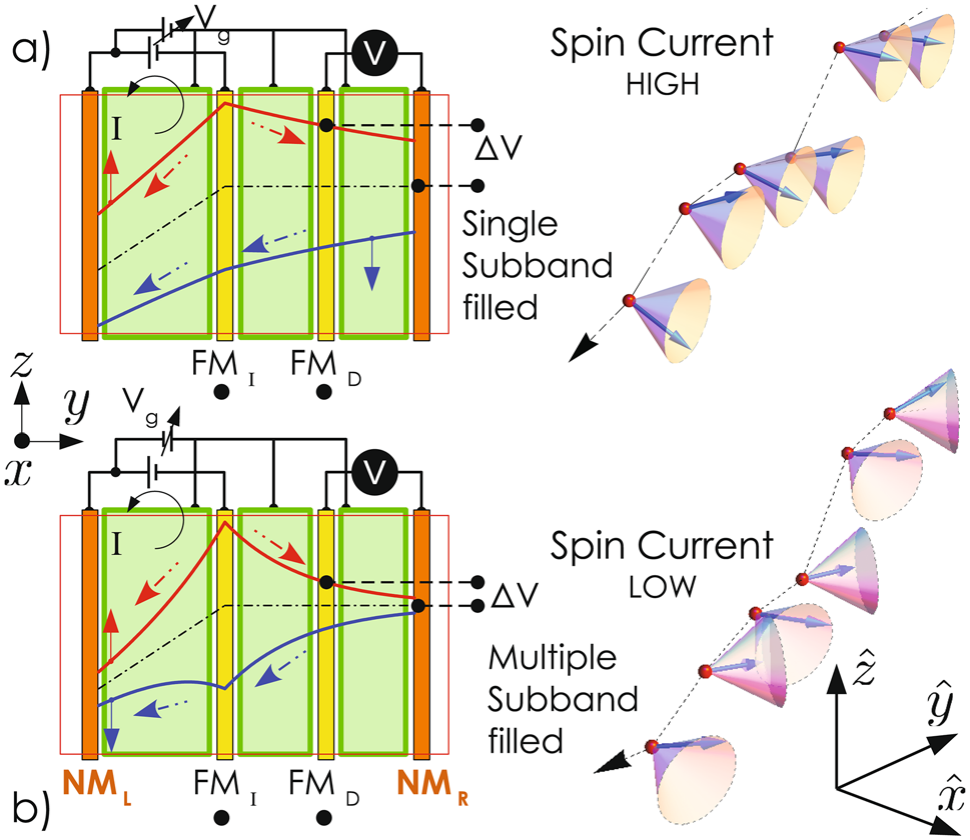
Schematic depiction of the variation of the spin-up (red curve), spin-down (blue curve), and average (dashed black curve) chemical potentials across the channel of the proposed spin-transistor. Broken arrows show the component of spin-resolved charge current. Red (blue) solid arrows represent $+(-)$*x* spin states. The green pads stand for the gate electrodes. When a single subband is filled, electron spin always precesses about ${\hat{x}}$ (+ or −) direction irrespective of any change in the direction of ${{\mathbf k}_{\mathbf {\parallel}}}$ due to scattering. On the other hand, when multiple subbands are filled, each scattering event results in a change in ${{\mathbf k}_{\mathbf {\parallel}}}$ that alters the direction of effective magnetic field and hence the spin precession axis.

Variation of $\mu$ and $\tau ^s_{y,z}$ with $r_{ch}$ are shown in Fig. 4c. As the gap between ${\mathbb {E}}$ and $E_f$ decreases with the increase of $r_{ch}$, $\mu$ reduces while $\tau ^s_{y,z}$ enhances. As obtained earlier, the coherence time ($\tau ^s_x$) for the spin projected along *x*-direction is infinite as far as DP mechanism (corrected up to $\sim {\text {O}}(k^3)$ term) is concerned . Relaxation of the *x*-component of spin should thus be governed by other processes such as Eliott-Yafet (EY) mechanism^29^. EY spin relaxation time can be estimated to be of the order of a few $\upmu$s in this case^42,43^. This assertion of zero rate of DP spin relaxation for *x* component of spin is strictly valid when a single subband is occupied. However, when more than one eigenstates are formed below the Fermi level, DP relaxation is activated even for the *x* projection of spin. This is because the wavefunction for the electrons near the Fermi level becomes a linear superposition of multiple eigenstates and $\langle k_x \rangle = 0$ is no longer holds^44,45^. Finite value of $\langle k_x \rangle$ results in a non-zero *y* component of ${\mathbf {\Omega }}$. Thus, $H_{SO}$ does not commute with any of the spin components resulting in a finite relaxation time even for the spin density $S_x$. This property can be exploited to envisage a different type of spin-transistor. A schematic of such a device is shown in Fig. 5a. In this nonlocal spin valve (spin-transistor) device, the pair of contacts in the middle is made of ferromagnetic metals (FM), while the contacts on edges are nonmagnetic (NM). The FM contact on the left is the spin injector (${\text {FM}}_{\text {I}}$) while the other works as the detector (${\text {FM}}_{\text {D}}$). Consider the case when both ${\text {FM}}_{\text {I}}$ and ${\text {FM}}_{\text {D}}$ are coupled to spin up (with respect to *x* axis) electrons. At an adequately high negative gate voltage, when only one subband is filled, the *x*-polarized spin up current injected from ${\text {FM}}_{\text {I}}$ reaches the detector electrode without losing its coherence. As a result, the spin current through the channel is high, and ‘spin-resolved charge voltage’ measured between ${\text {FM}}_{\text {D}}$ and ${\text {NM}}_{\text {R}}$ will be sufficiently high. Upon application of a large positive gate bias, more than one subband are filled. At this condition, the direction of the effective magnetic field (${\mathbf {\Omega }}$), which is the Larmor precession axis of electron spin, get randomized due to successive scattering events. As a result, the *x* projection of spin also relaxes. This should result in a rapid decay of pure spin current in the channel between ${\text {FM}}_{\text {I}}$ and ${\text {FM}}_{\text {D}}$, as depicted in Fig. 5b. The device can thus act as a spin-transistor. Note that, in most of the spin-transistor proposals, a control over spin relaxation is achieved through electric field-induced change in the effective magnetic field experienced by the carriers in the channel^46,47^. Here, the goal can be achieved by changing the carrier concentration in the channel, which can turn the DP mechanism on/off by removing/introducing additional eigenstates below the Fermi level^14,44,45^. As mentioned before that the nature of quantum confinement in this system is such that it preserves the symmetry between conduction and valence band everywhere irrespective of the fact whether the nanowedge is geometrically symmetric or not. This results in the suppression of the Rashba effect, which comes as an added advantage in maintaining the spin coherence of electrons in this system. Note that the present 2DEG system is substantially different from the 2DEG, formed at the heterojunction of *a*-directional GaN/AlGaN as discussed in “Supplementary”.

## Conclusions

Spin relaxation of electrons in a 2DEG formed at the central vertical plane of a *c*-oriented wedge-shaped WZ-GaN nanowall is theoretically investigated. It has been found that the component of spin projected in the plane of the confinement relaxes through DP mechanism with a time-scale of a few tens of picoseconds everywhere in the channel, while the spin component along the direction of confinement never relaxes through DP process in a part of the channel, where the Fermi-level occupies only one subband. However, by applying appropriate positive gate bias, electron concentration in the well can be sufficiently enhanced, which can bring in more than one subband below the Fermi-level in most of the channel. In this situation, DP relaxation mechanism is switched-on for the spin component along the confinement direction. One can envisage a spin-transistor, where this property can be exploited to electrically manipulate the spin current.

## Supplementary Information

Supplementary Information.

## References

[1] Datta S, Das B (1990). Electronic analog of the electro-optic modulator. Appl. Phys. Lett..

[2] Salis G (2001). Electrical control of spin coherence in semiconductor nanostructures. Nature.

[3] Hernández-Mínguez A, Biermann K, Hey R, Santos PV (2016). Electric control of spin transport in GaAs (111) quantum wells. Phys. Rev. B.

[4] Trier, F. *et al.* Electric-field control of spin current generation and detection in ferromagnet-free SrTiO_3_-based nanodevices. *Nano Lett*. **20**, 395 (2019). 10.1021/acs.nanolett.9b04079 31859513

[5] Jena D (2004). Spin scattering by dislocations in III–V semiconductors. Phys. Rev. B.

[6] Panda J, Ramu M, Karis O, Sarkar T, Kamalakar MV (2020). Ultimate spin currents in commercial chemical vapor deposited graphene. ACS Nano.

[7] Serrano IG (2019). Two-dimensional exible high diffusive spin circuits. Nano Lett..

[8] Dyakonov, M.I. Spin Physics in Semiconductors, Vol. 157, 1–532. Springer Series in Solid-State Sciences, Springer International Publishing (2008).

[9] Harmon NJ, Putikka WO, Joynt R (2009). Theory of electron spin relaxation in ZnO. Phys. Rev. B.

[10] Buß JH, Rudolph J, Natali F, Semond F, Hägele D (2010). Temperature dependence of electron spin relaxation in bulk GaN. Phys. Rev. B.

[11] Rudolph J, Buß JH, Hägele D (2014). Electron spin dynamics in GaN. Physica Status Solidi (b).

[12] Litvinov VI (2006). Polarization-induced rashba spin-orbit coupling in structurally symmetric III-Nitride quantum wells. Appl. Phys. Lett..

[13] Besbas J (2010). Spin relaxation of free excitons in narrow GaN/Al_x_Ga_1–x_N quantum wells. Phys. Rev. B.

[14] Buß JH, Fernndez-Garrido S, Brandt O, Hägele D, Rudolph J (2019). Electron spin dynamics in mesoscopic GaN nanowires. Appl. Phys. Lett..

[15] Park T-E, Park YH, Lee J-M, Kim SW, Park HG, Min B-C, Kim H, Koo HC, Choi H-J, Han SH, Johnson M, Chang J (2017). Large spin accumulation and crystallographic dependence of spin transport in single crystal gallium nitride nanowires. Nat. Commun..

[16] Kum H, Heo J, Jahangir S, Banerjee A, Guo W, Bhattacharya P (2012). Room temperature single gan nanowire spin valves with FeCo/MgO tunnel contacts. Appl. Phys. Lett..

[17] Kammermeier M, Seith A, Wenk P, Schliemann J (2020). Persistent spin textures and currents in wurtzite nanowire-based quantum structures. Phys. Rev. B.

[18] Chen J-Y, Wong T-M, Chang C-W, Dong C-Y, Chen Y-F (2014). Self-polarized spin-nanolasers. Nat. Nanotechnol..

[19] Szafrański M (1990). Microscopic origin of spontaneous polarization and absolute sense of pyroelectric and piezoelectric coeffcients in α-Liio3. Solid State Commun..

[20] Kong XY, Wang ZL (2003). Spontaneous polarization-induced nanohelixes, nanosprings, and nanorings of piezoelectric nanobelts. Nano Lett..

[21] Deb S, Bhasker HP, Thakur V, Shivaprasad SM, Dhar S (2016). Polarization induced two dimensional confinement of carriers in wedge shaped polar semiconductors. Sci. Rep..

[22] Deb S, Dhar S (2018). Wedge-shaped GaN nanowalls: A potential candidate for two-dimensional electronics and spintronics. Spin.

[23] Bhasker HP, Dhar S, Sain A, Kesaria M, Shivaprasad SM (2012). High electron mobility through the edge states in random networks of c-axis oriented wedge-shaped GaN nanowalls grown by molecular beam epitaxy. Appl. Phys. Lett..

[24] Bhasker HP, Thakur V, Shivaprasad SM, Dhar S (2015). Quantum coherence of electrons in random networks of c-axis oriented wedge-shaped GaN nanowalls grown by molecular beam epitaxy. J. Phys. D: Appl. Phys..

[25] Bhasker HP, Thakur V, Shivaprasad SM, Dhar S (2015). Role of quantum confinement in giving rise to high electron mobility in GaN nanowall networks. Solid State Commun..

[26] Bhasker HP, Thakur V, Kesaria M, Shivaprasad SM, Dhar S (2014). Transport and optical properties of c-axis oriented wedge shaped GaN nanowall network grown by molecular beam epitaxy. AIP Conf. Proc..

[27] Jain K, Chakraborti H, Joshi BP, Pal B, Monish M, Shivaprasad SM, Dhar S, Gupta KD (2019). Effect of invasive probes on measurement of magneto-transport in macroscopic samples: A gallium nitride case study. J. Appl. Phys..

[28] Chakraborti H, Deb S, Schott R, Thakur V, Chatterjee A, Yadav S, Saroj RK, Wieck A, Shivaprasad SM, Gupta KD, Dhar S (2018). Coherent transmission of superconducting carriers through a ~ 2 μm polar semiconductor. Supercond. Sci. Technol..

[29] Wu MW, Jiang JH, Weng MQ (2010). Spin dynamics in semiconductors. Phys. Rep..

[30] Jiang XH, Shi JJ, Zhang M, Zhong HX, Huang P, Ding YM, Yu TJ, Shen B, Lu J, Wang X (2014). Enhancement of TE polarized light extraction e_ciency in nanoscale AlNm/GaNn(m > n) superlattice substitution for Al-rich AlGaN disorder alloy: ultra-thin GaN layer modulation. N. J. Phys..

[31] Fu JY, Wu MW (2008). Spin-orbit coupling in bulk ZnO and GaN. J. Appl. Phys..

[32] Fu J, Penteado PH, Candido DR, Ferreira GJ, Pires DP, Bernardes E, Egues JC (2020). Spin–orbit coupling in wurtzite heterostructures. Phys. Rev. B.

[33] Kainz J, Rössler U, Winkler R (2003). Anisotropic spin-splitting and spin-relaxation in asymmetric zinc blende semiconductor quantum structures. Phys. Rev. B.

[34] Kainz J, Rössler U, Winkler R (2004). Temperature dependence of D'yakonov-Perel' spin relaxation in zinc-blende semiconductor quantum structures. Phys. Rev. B.

[35] Cartoixà X, Ting DZ-Y, Chang Y-C (2005). Suppression of the D'yakonov-Perel' spinrelaxation mechanism for all spin components in [111] zincblende quantum wells. Phys. Rev. B.

[36] Fabian, J., Matos-Abiague, A., Ertler, C., Stano, P. & Uti, P. Semiconductor spintronics. *Acta Physica Slovaca* **57**(4–5), 565 (2007).

[37] Averkiev NS, Golub LE, Willander M (2002). Spin relaxation anisotropy in two-dimensional semiconductor systems. J. Phys. Condens. Matter.

[38] Harmon NJ, Putikka WO, Joynt R (2011). Prediction of extremely long mobile electron spin lifetimes at room temperature in wurtzite semiconductor quantum wells. Appl. Phys. Lett..

[39] Lo I, Gau MH, Tsai JK, Chen YL, Chang ZJ, Wang WT, Chiang J-C, Aggerstam T, Lourdudoss S (2007). Anomalous k-dependent spin splitting in wurtzite Al_x_Ga_1__−__x_N/GaN heterostructures. Phys. Rev. B.

[40] Wang W-T, Wu CL, Tsay SF, Gau MH, Lo I, Kao HF, Jang DJ, Chiang J-C, Lee M-E, Chang Y-C (2007). Dresselhaus effect in bulk wurtzite materials. Appl. Phys. Lett..

[41] Kapon E, Gossard AC (1994). Lateral patterning of quantum well heterostructures by growth on nonplanar substrates. Epitaxial Microstructures, Volume 40 of Semiconductors and Semimetals.

[42] Pikus, G. E. & Titkov, A. N. Optical orientation, Vol 8, 1–523 in (eds Meier, F., Zakharchenya, B. P.) (North-Holland Physics Publishing, Elsevier Science Publishers, 1984).

[43] Buß JH, Schupp T, As DJ, Brandt O, Hägele D, Rudolph J (2016). Electron spin dynamics in cubic gan. Phys. Rev. B.

[44] Lu C, Schneider HC, Wu MW (2009). Electron spin relaxation in n-type InAs quantum wires. J. Appl. Phys..

[45] Döhrmann S, Hägele D, Rudolph J, Bichler M, Schuh D, Oestreich M (2004). Anomalous spin dephasing in (110) gaas quantum wells: Anisotropy and intersubband effects. Phys. Rev. Lett..

[46] Chuang P, Ho S-C, Smith LW, Sfigakis F, Pepper M, Chen C-H, Fan J-C, Griffiths JP, Farrer I, Beere HE, Jones GAC, Ritchie DA, Chen T-M (2015). All-electric all-semiconductor spin field-effect transistors. Nat. Nanotechnol..

[47] Park YH, Choi JW, Kim H-J, Chang J, Han SH, Choi H-J, Koo HC (2017). Complementary spin transistor using a quantum well channel. Sci. Rep..

