# Supplementary material for: Spin transport in polarization induced two-dimensional electron gas channel in c-GaN nano-wedges
Source: Sci Rep. 2021 Mar 5;11:5277. doi: 10.1038/s41598-021-84451-y (PMC7935858; doi:10.1038/s41598-021-84451-y)
Supplement: Supplementary file 1 — Supplementary Information. [file 41598_2021_84451_MOESM1_ESM.pdf]

Supplementary Information

Spin transport in polarization induced two dimensional electron gas channel in c-GaN nano-wedges

Swarup Deb, Subhabrata Dhar

Department of Physics, Indian Institute of Technology Bombay, Powai, Mumbai-400076, India

S1. Derivation of spin relaxation time

In order to calculate the DP spin relaxation time we follow the formalism described by N. S. Averkiev *et al.*[1] and J. Fabian *et al.*[2]. The DP relaxation rate for spin density, $S_i(t)$ projected along $\hat{i}$ ($i = x, y, z$) can be written as[3]:

$$\dot{S}_i(t) = -\frac{1}{2\hbar^2}\sum_{n=-\infty}^{n=\infty}\frac{\int_0^\infty d\mathcal{E}(\vec{k}_{\shortparallel})\delta f \tau_n Tr([H_{-n},[H_n,\sigma_j]]\sigma_i)}{\int_0^\infty d\mathcal{E}(\vec{k}_{\shortparallel})\delta f}S_j(t) \tag{S1}$$

where, δf=$(f_+ - f_-)$, $f_\pm$ are the Fermi distribution functions for electrons with spin $\pm 1/2$. τ_n are the relaxation times[3, 4], which can be expressed as:

$$\tau_n^{-1}(k_{\shortparallel}) = \frac{\mathcal{A}}{4\pi^2}\int_0^{2\pi}\mathcal{S}(\vec{k}_{\shortparallel},\vec{k}'_{\shortparallel})[1-cos(n\theta)]d\theta \tag{S2}$$

where, $\mathcal{S}(\vec{k}_{\shortparallel},\vec{k}'_{\shortparallel})$ the spin independent momentum scattering rate between $\vec{k}_{\shortparallel}$ and $\vec{k}'_{\shortparallel}$, θ the angle between the initial and final wave vectors, $\mathcal{A}$ the box normalization factor for the free part of the wave function of the confined electrons. H_n are the Fourier harmonics of spin-orbit Hamiltonian:

$$H_n = \int_0^{2\pi}\frac{d\phi}{2\pi}H_{SO}e^{-\mathrm{i}n\phi} \tag{S3}$$

Because of the presence of linear and cubic terms of $k_{\shortparallel}$ in H_{SO}, H_n survives only for $n = \pm 3$ and ± 1.

It can be shown that:

$$\begin{aligned} H_{+1} = H_{-1} &= (\alpha_R - \beta_D\langle k_x^2\rangle)/2\, k\sigma_x + \beta_D b_D/8\, k^3\sigma_x - 3\beta_D/8\, k^3\sigma_x \\ &= C_1 k\sigma_x + C_2 k^3\sigma_x \end{aligned} \tag{S4}$$

$$\begin{aligned} H_{+3} = H_{-3} &= -\beta_D b_D/8\, k^3\sigma_x - \beta_D/8\, k^3\sigma_x \\ &= C_3 k^3\sigma_x \end{aligned} \tag{S5}$$

For, $\dot{S}_x(t)$ the trace, $Tr(\cdots)$ in equation (S1) takes the form:

$$Tr([H_{-n},[H_n,\sigma_j]]\sigma_x) \propto Tr([\sigma_x,[\sigma_x,\sigma_j]]\sigma_x);\ j=x,y,z \tag{S6}$$

In the above relation, the $k_{\shortparallel}$ dependent prefactors are dropped for simplicity. We have also taken the advantage of the fact that in such quantum confined system H_{SO} get coupled to σ_x, only. The Pauli matrices follow the commutation relation $[\sigma_i,\sigma_j] = 2\mathtt{i}\xi_{ijk}\sigma_k$, ξ_{ijk} is the Levi-Civita tensor. Using this relation the trace can be evaluated as:

$$\begin{aligned} Tr([\sigma_x,[\sigma_x,\sigma_j]]\sigma_x) &= 2\mathtt{i}Tr([\sigma_x,\xi_{xjk}\sigma_k]\sigma_x) \\ &= -4\xi_{xjk}\xi_{xkj}Tr(\sigma_j\sigma_x) \end{aligned} \tag{S7}$$

Again, $Tr(\sigma_j\sigma_x) \neq 0$ only if $j = x$ but in that case ξ_{xjk} or ξ_{xkj} are 0 and vice versa. Thus the $Tr(\cdots)$ term in the numerator of equation (S1) becomes 0 for $\dot{S}_x(t)$, leading to

$$\dot{S}_x(t) = 0 \tag{S8}$$

Derivation of $\dot{S}_y$ and $\dot{S}_z$ is slightly tedious. The trace in the numerator of equation (S1) has to be evaluated for all possible combination of i, j and n. As an example we evaluate the terms for $\dot{S}_y$ in table S1. Thus i has been replaced with y.

TABLE S1: Evaluating "$\tau_n Tr([H_{-n},[H_n,\sigma_j]]\sigma_i)S_j(t)$" from equation (S1) as $i = y$

n	j=x	j=y	j=z
+3	$\tau_3 Tr([H_{-3},[H_3,\sigma_x]]\sigma_y)S_x(t)$	$\tau_3 Tr([H_{-3},[H_3,\sigma_y]]\sigma_y)S_y(t)$	$\tau_3 Tr([H_{-3},[H_3,\sigma_z]]\sigma_y)S_z(t)$
	$= 0$	$= \tau_3 8C_3^3k^6S_y(t)$	$= 0$
+1	$\tau_1 Tr([H_{-1},[H_1,\sigma_x]]\sigma_y)S_x(t)$	$\tau_1 Tr([H_{-1},[H_1,\sigma_y]]\sigma_y)S_y(t)$	$\tau_1 Tr([H_{-1},[H_1,\sigma_z]]\sigma_y)S_z(t)$
	$= 0$	$= \tau_1 8(C_1k + C_2k^3)^2S_y(t)$	$= 0$
-1	$\tau_{-1} Tr([H_1,[H_-1,\sigma_x]]\sigma_y)S_x(t)$	$\tau_{-1} Tr([H_1,[H_{-1},\sigma_y]]\sigma_y)S_y(t)$	$\tau_{-1} Tr([H_1,[H_{-1},\sigma_z]]\sigma_y)S_z(t)$
	$= 0$	$= \tau_{-1} 8(C_1k + C_2k^3)^2S_y(t)$	$= 0$
-3	$\tau_{-3} Tr([H_3,[H_-3,\sigma_x]]\sigma_y)S_x(t)$	$\tau_{-3} Tr([H_3,[H_{-3},\sigma_y]]\sigma_y)S_y(t)$	$\tau_{-3} Tr([H_3,[H_{-3},\sigma_z]]\sigma_y)S_z(t)$
	$= 0$	$= \tau_{-3} 8C_3^3k^6S_y(t)$	$= 0$

$\dot{S}_z$ can also be evaluated in a similar fashion. It is important to note that $\dot{S}_y$ only depends on the y spin projection, S_y. The same hold for the z component also. Thus one can write the spin relaxation time of y and z components as:

$$\frac{\dot{S}_y(t)}{S_y(t)} = -\frac{1}{\tau_y^s}; \text{ and } \frac{\dot{S}_z(t)}{S_z(t)} = -\frac{1}{\tau_z^s} \tag{S9}$$

Following reference[1], we write τ_y^s and τ_z^s as

$$\frac{1}{\tau_y^s} = \frac{1}{\tau_z^s} = \frac{1}{\tau_{y,z}^s} = \frac{1}{2\hbar^2}\left[\sum_{n=-1,1} 8(C_1 k_{\shortparallel} + C_2 k_{\shortparallel}^3)^2 \tau_n + \sum_{n=-3,3} 8C_3^2 k_{\shortparallel}^6 \tau_n\right] \tag{S10}$$

Following parameter values are used during numerical calculation: α_R=9.0 meVÅ; b_D=3.959; β_D=0.32 eVÅ^3[5]

S2. Spin dynamics:*a*-plane 2DEG in a AlGaN/GaN Heterojunction versus *a*-plane 2DEG in *c*-oriented GaN nanowedge

At a first glance, 2D electron gas confined in a-plane of AlGaN/GaN[6] appears very similar to the present case of the 2DEG formed in a c-oriented wedge shaped GaN nanowall structure. However, unlike the present case, such a heterojunction lacks spatial inversion symmetry(SIA) leading to the existence of a net electric field. This assertion requires a more careful discussion.

In a quantum confined system, the effective electric field can be quantified by the expectation value of the field *i.e.* $-\langle \frac{\partial E_c}{\partial x} \rangle / q_e$. Where, E_c is the conduction band energy profile in real space and q_e is the electron charge. Using the identities from Ehrenfest theorem:

$$\langle -\frac{\partial E_c}{\partial x} \rangle = \frac{d}{dt}\langle p \rangle \tag{S11}$$

$$= \frac{d}{dt}\left(m\frac{d}{dt}\langle x \rangle\right) \tag{S12}$$

For a bound eigen state, time derivative of $\langle x \rangle = 0$ and hence the effective electric field is also zero. This was first pointed out by T. Ando[7, 8] who argued that the spin splitting of conduction band must be negligibly small in such system. Later, Lassnig[9] showed that Rashba field in such quantum well system will be proportional to the average electric field of valence band seen by the conduction band electrons *i.e.* $\int |\psi_c|^2 \frac{\partial E_v}{\partial x} dx$. Where, ψ_c denotes the wave-function for the electrons at the conduction band edge and E_v is the valence band edge profile. In a a-plane 2DEG of AlGaN/GaN heterojunction, the band offset breaks

the symmetry between the conduction and valence band across the junction. As a result, $\int|\psi_c|^2\frac{\partial E_v}{\partial x}dx$ does not vanish for a bound state while $\int|\psi_c|^2\frac{\partial E_c}{\partial x}dx$ does. Due to Rashba effect, this average field gives rise to an effective magnetic field acting perpendicular to $\hat{x}$[8, 10] and H_{SO} becomes

$$H_{SO}(\vec{k}) = \{\alpha_R+\beta_D(b_D k_z^2-\langle k_x^2\rangle-k_y^2)\}\begin{pmatrix} k_y & 0 & 0 \end{pmatrix}\begin{pmatrix}\sigma_x\\ \sigma_y\\ \sigma_z\end{pmatrix}+\alpha_x\begin{pmatrix} 0 & k_z & -k_y \end{pmatrix}\begin{pmatrix}\sigma_x\\ \sigma_y\\ \sigma_z\end{pmatrix} \quad \text{(S13)}$$

Clearly, H_{SO} does not commute with any of the σ's. Spin density S thus follows a different dynamics than in the present case. It should be noted that in case of the a-plane 2DEG in c-oriented wedge shaped GaN nanowalls, both $\langle\psi_c|\frac{\partial E_c}{\partial x}|\psi_c\rangle$=$\langle\psi_c|\frac{\partial E_v}{\partial x}|\psi_c\rangle$=0 as the symmetry between the conduction and valence band across the junction is intact. This is true even when the shape of the wedge is asymmetric. Therefore, the spin dynamics remains to be the same for both asymmetric and symmetric nanowedges.

---

[1] N S Averkiev, L E Golub, and M Willander. Spin relaxation anisotropy in two-dimensional semiconductor systems. *Journal of Physics: Condensed Matter*, 14:R271, 2002.

[2] Jaroslav Fabian, Alex Matos-Abiague, Christian Ertler, Peter Stano, and Igor uti. Semiconductor spintronics. *Acta Physica Slovaca. Reviews and Tutorials*, 57(4-5), Aug 2007.

[3] N. J. Harmon, W. O. Putikka, and Robert Joynt. Prediction of extremely long mobile electron spin lifetimes at room temperature in wurtzite semiconductor quantum wells. *Appl. Phys. Lett.*, 98:073108, 2011.

[4] J. Kainz, U. Rössler, and R. Winkler. Temperature dependence of dyakonov-perel spin relaxation in zinc-blende semiconductor quantum structures. *Phys. Rev. B*, 70:195322, 2004.

[5] Jörg Rudolph, Jan Heye Buß, and Daniel Hgele. Electron spin dynamics in GaN. *physica status solidi (b)*, 251:1850, 2014.

[6] Yasuhiro Isobe, Hiromichi Ikki, Tatsuyuki Sakakibara, Motoaki Iwaya, Tetsuya Takeuchi, Satoshi Kamiyama, Isamu Akasaki, Takayuki Sugiyama, Hiroshi Amano, Mamoru Imade,

Yasuo Kitaoka, and Yusuke Mori. Nonpolara-plane AlGaN/GaN heterostructure field-effect transistors grown on freestanding GaN substrate. *Applied Physics Express*, 4:064102, 2011.

[7] Takashi Matsuda and Kanji Yoh. Enhancement of spin–orbit interaction by bandgap engineering in inas-based heterostructures. *J. Electron. Mater.*, 37:1806, 2008.

[8] P. S. Eldridge, W. J. H. Leyland, P. G. Lagoudakis, R. T. Harley, R. T. Phillips, R. Winkler, M. Henini, and D. Taylor. Rashba spin-splitting of electrons in asymmetric quantum wells. *Physical Review B*, 82:045317, 2010.

[9] R. Lassnig. k→·p→ theory, effective-mass approach, and spin splitting for two-dimensional electrons in GaAs-GaAlAs heterostructures. *Phys. Rev. B*, 31:8076, 1985.

[10] P. S. Eldridge, W. J. H. Leyland, J. D. Mar, P. G. Lagoudakis, R. Winkler, O. Z. Karimov, M. Henini, D. Taylor, R. T. Phillips, and R. T. Harley. Rashba conduction band spin-splitting for asymmetric quantum well potentials. *Journal of Superconductivity and Novel Magnetism*, 23:157, 2009.
